# Religious accommodation, agonism, agnosticism in healthcare: A commentary on Joshua Hordern, ‘Accommodating religion and belief in healthcare: Political threats, agonistic democracy and established religion’

**DOI:** 10.1111/bioe.13120

**Published:** 2022-11-30

**Authors:** Dominic J. C. Wilkinson

**Affiliations:** ^1^ Oxford Uehiro Centre for Practical Ethics, Faculty of Philosophy University of Oxford Oxford UK; ^2^ Newborn Care John Radcliffe Hospital Oxford UK; ^3^ Murdoch Children's Research Institute Melbourne Australia

## 1 ‘ACCOMMODATION’

Consider the following examples

1. Space competition. A publicly funded hospital is finalising plans for its new building. There is intense pressure and competition between departments for the finite physical space and budget. One question relates to how much space is set aside within the new building for a ‘prayer space’. Should there be separate spaces for different religions or a common space for those of different religions? Should the allocation of this space come out of that set aside for clinical areas or out of those set aside for patients and visitors (e.g., patient lounges/cafeteria)?^1^
2. Resource competition. During the coronavirus pandemic, some critically ill patients were treated with a highly scarce and resource‐intensive form of treatment—extra‐corporeal membrane oxygenation (ECMO). This provides temporary support for patients with life‐threatening hypoxic respiratory failure who may otherwise be highly likely to die. In cases where patients deteriorate or fail to improve over a period of time (e.g., >2 weeks), it would be standard practice to withdraw ECMO. However, some families refuse permission for withdrawal of treatment, citing the religious beliefs of the patient. Should scarce treatment be continued (for a longer period or indefinitely) in patients with particular religious beliefs?^2^

Cases like that of Space Competition and Resource Competition give rise to questions about reasonable accommodation within public healthcare systems like the National Health Service. In particular, they require us to address whether such systems should accommodate religious views, and if so, *how much*?

In his paper ‘Accommodating religion and belief in healthcare’, Joshua Hordern starts by questioning whether the language of ‘accommodation’ is itself problematic—containing an implicit framing assumption that the default is for patients not to be religious.^3^ He cites and endorses the worry expressed by Lori Beaman,^4^ that this risks automatically biasing discourse against those who are religious by implying that some people's religious views create *additional* or *extra‐ordinary* demands on the system over and above the needs of ‘normal’ (nonreligious) members of the community.

Do we need to rethink the terminology of accommodation? Some concepts in this area are ethically thicker than others. The idea of ‘Tolerance’ has long been criticised, even as it has been widely promoted and encouraged in political discourse. The concern is that it conceals an attitude of disagreement or dislike. Goethe claimed that ‘To tolerate means to insult’.^5^ Although there can be different ways of understanding the concept, including versions (‘respect tolerance’ or ‘esteem tolerance’) that do not involve any devaluation of those who are tolerated, it may be better to avoid the language of religious ‘tolerance’.

In contrast, when it comes to accommodation, pace Beaman, there is no obvious necessary negative connotation. The word derives from Latin ‘accommodare’—to fit one thing to another. Its literal meanings include the notion of adaptation or adjustment, but also of making space for, assisting, providing comfort or convenience. It does not (to my mind) problematically bias debate about allocation of resources to talk about religious accommodation. But of course words can have different received meanings from their intended meanings. And they may be understood in very different ways depending on people's backgrounds.

Hordern, ultimately, concedes the ‘positive intent’ of the language of accommodation.^6^ More important than the terminology, however, is what we take accommodation to mean in practice and how we enact it. That is the focus of the majority of Hordern's paper.

## 2 AGONISM

Hordern suggests that the practice of accommodation should be modified to include ideas of agonism and deep equality that he draws from Beaman's work (and from the political philosophy of Jonathan Chaplin). What would this mean for cases like Space Competition or Resource Competition?

In his paper, Hordern does not provide a clear answer to this. Partly this is because, as he admits, Beaman's own work does not delve deeply into conceptual debates about religion in public life, and consequently, has a lack of ‘focus on the sharp end of decision‐making difficulties in public institutions’.^7^ However, if we are to determine whether we need a different approach to accommodation in healthcare, it would be important to try to ascertain what this approach would mean.

‘Deep equality’ is a concept that Beaman wrote about in her book … According to Beaman, it is:

a vision of equality that … relocates equality as a process rather than a definition, and as lived rather than prescribed. It recognizes equality as an achievement of day‐to‐day interaction, and is traceable through agonistic respect, recognition of similarity, and a concomitant acceptance of difference, creation of community, and neighbourliness^8^

Agonism, by contrast, emerged in the 1990s as a specific post‐modernist response to political liberalism. When thinking about how to structure our political institutions, rather than setting aside or bracketing elements of citizens’ core beliefs (as Rawlsian liberalism suggests), *agonism* sees value‐based conflict as a vital and valuable part of democracy.^9^ Beaman cites the political philosopher William Connolly:

An ethos of agonistic respect grows out of mutual appreciation for the ubiquity of faith and the inability of contending parties, to date, to demonstrate the truth of one faith over live candidates… The relation is agonistic in two senses: you absorb the agony of having elements of your own faith called into question by others, and you fold agonistic contestation of others into the respect that you convey toward them.^10^

Both of these ideas focus on the *process* of accommodation rather than the *substance* of decision‐making. They concentrate on the mindset that underlies interactions. Because of this, it can be difficult to know exactly what they would mean for cases like the ones described at the start of the paper.

One possibility, suggested by the reference to ‘deep *equality*’, is that the ethical answer to questions of accommodation should be fundamentally egalitarian. From such a perspective, we might want ensure that all members of society (religious or nonreligious) have equal access to space for personal reflection or prayer^11^ or equal access to treatment like ECMO. For example, this might include some sort of allocation of physical space within the hospital in proportion to the demographics in the community. (In London, this might mean 49% of space allocated to Christians, 25% to those with no religion, 14% to Muslims, 5% to Hindus).^12^ For allocation of a treatment like ECMO, it might yield a lottery for allocation.^13^

However, it is not clear that egalitarianism is the right answer to religious diversity and questions of accommodation. For one thing, it would very significantly disadvantage those from minority religious groups (or nonreligious minorities in highly religious countries). They would potentially end up with very limited or no allocation of resources like space within a hospital. On the other hand, allocation of treatment on a random basis could lead to significant unfairness in distribution that unfairly favours some. (If some patients will not accept withdrawal of treatment, they may consequently receive a much longer duration of treatments like ECMO, which would come at the cost of several other patients who are thereby denied access to treatment).^14^ Next, a simple egalitarian approach would potentially promote equality at the cost of *equity*. When thinking either about allocation of space within a hospital or a treatment like ECMO, patients’ different religious backgrounds might generate very different needs for the space/treatment.

In fact, it seems that neither Hordern nor Beaman would wish to endorse a simplistic egalitarian approach. Deep equality and agonistic respect are all about engaging with the views and values of persons—recognising and responding to their differences, rather than mechanically treating them as all alike. However, it is also tempting to note that agonistic respect is easy when resources are unlimited. If there were no limit to the space available in the new hospital building, we could simply incorporate as much space as needed by any patient who might use the facility. If there were no limit to the availability of ECMO, there would be no (resource‐based) reason to be concerned about continuing treatment for a prolonged period. But resources are finite. What is needed is some way of arbitrating fairly between different claims.

## 3 AGNOSTICISM

Part of the idea of reasonable accommodation is that it would attempt to weigh up and impartially decide how much weight to give to the competing claims of different individuals or different groups. How should we go about doing that?

Hordern is critical of one particular approach—that informed by Rawlsian political liberalism. His concern (one shared by Connolly) is that the account of public reason in this political philosophy potentially excludes religious values from debate. There are two separate strands to his concern: first, that this way of thinking about reasonable accommodation biases debate (and allocation) in favour of secular perspectives and second, that it damages communication by requiring those who are religious to self‐censor and express their views in a form of ‘secular esperanto’.^15^

The first of these concerns is important. I agree with Hordern that when thinking about questions like Space Competition or Resource Competition, our starting assumption should be agnostic, not atheist. The alternative would beg a substantive question about the rightness of one particular perspective. However, it is worth noting three elements of reasonable accommodation even if we take such a neutral ‘view from nowhere’ as our starting point.^16^ First, when we are considering different competing claims, an agnostic approach should not give special weight to particular views or preferences simply because they are ‘religious’. It would discriminate against the nonreligious to allow prolonged treatment with ECMO for religious patients, but not to those who wish for such treatment for non‐religious reasons. Second, some reasons have wider traction than others. At least some non‐religious reasons (e.g., about the value of health, the importance of treating or preventing suffering) are widely shared in a way that some religious reasons are not (e.g., belief in the power of prayer or the possibility of miracles).^17^ Those shared reasons may be given particular weight when we are making decisions. Third, that when we are thinking about allocating scarce resources, we may not wish to privilege particular points of view. But we can and should note that some views are more resource‐demanding than others. In considering the allocation of prayer space within a public hospital, adherents of some religions are accepting of a shared multifaith space, while others may reject that and wish for a dedicated area.^18^ Whether that can be accommodated or not will depend on how much space is available and how we balance the needs of different groups. But it is relevant to allocation that respecting certain views would give those individuals a greater share of a scarce resource than others.

Hordern's second concern leads to his call for civility in discourse within pluralist democracies and within healthcare institutions. Here, there is little to disagree with. Patients and health professionals *should* be free to express themselves in terms of ‘a *full* range of reasons … including those which, though perhaps intelligible, might be unpersuasive in the sense of others coming to see the world differently’.^19^ If that is what is required for a more agonistic approach to accommodation, then we should indeed embrace this model of communication and pluralist discourse. However, we might also note another seemingly inevitable implication of agonism that may not be so warmly received. Religious patients and groups must ‘absorb the agony of having elements of [their] own faith called into question by others’.^20^ They must also come to terms with the inevitability that in a resource‐constrained publicly funded healthcare system, their religious preferences for treatment provision (no matter how deeply or sincerely held) cannot always be met.

## CONFLICT OF INTEREST

The author declares no conflict of interest.

## Footnotes

1 Sophie Gilliat Ray describes some of the tensions arising between religious groups when spaces are shared. She also notes some problems arising from creating a separate space for Muslim prayer, ‘if they have a prayer room, we must have one too’. Gilliat‐Ray, S. (2005). From ‘chapel’ to ‘prayer Room’: The production, use, and politics of sacred space in public institutions. *Culture and Religion*, *6*, 287–308: 299. See also Stammers, T., & Bullivant, S. (2012). Secularism. In M. Cobb, C. M. Puchalski, & B. Rumbold (Eds.), *Oxford textbook of spirituality in healthcare.* Oxford University Press.

2 In February 2021, the Court of Protection heard a case of N, a 32‐year‐old who had deteriorated on ECMO. Her family would not support withdrawal of ECMO. The judge noted that ‘this is not grounded in any real difference as to the medical situation; it is, for them, a conflict between a religious belief, genuinely and devoutly held, and medical science. It is a conflict which cannot be reconciled. To condone any act that would be seen to bring life to an end would, the family believe, be inconsistent with their faith’. NZ, Re (Mental Capacity Act 2005) [2021] EWCOP 16 (23 February 2021) In a very similar court case, a 52‐year‐old Pentecostal Christian, K., was on ECMO for 3.5 months, and his family believed that there was a chance of recovery with prayer and would not agree to withdrawal of treatment. KM, Re [2021] EWCOP 42 (10 May 2021).

3 Hordern, J. (2023). Accommodating religion and belief in healthcare: Political threats, agonistic democracy and established religion. *Bioethics*, 37 (1), 15–27.

4 Beaman, L. G. (2017). *Deep equality in an era of religious diversity* (1st ed.). Oxford University Press.

5 Goethe 1829 Maximen und Reflexionen cited in Forst, R. (2017). Toleration. In E. Zalta (Ed.), *The Stanford Encyclopedia of Philosophy* (Fall 2017 ed.). https://plato.stanford.edu/archives/fall2017/entries/toleration/

6 Hordern, op. cit. note 3, p. 3.

7 Ibid: 4.

8 Beaman, op. cit. note 4, p. 13.

9 Deveaux, M. (1999). Agonism and pluralism. *Philosophy & Social Criticism*, *25*, 1–22: 2.

10 Connolly, W. E. (2005). *Pluralism* (p. 124). Duke University Press.

11 Stammers, op. cit. note 1.

12 Population by religion, borough. (2018). Retrieved November 23, 2021, from https://data.london.gov.uk/dataset/percentage-population-religion-borough?resource=abfb6175-f489-4c6e-add2-f4d323183224. An alternative might be to have a multifaith space available at different times of the day in proportion to the demographics of the population.

13 Dao, B., Savulescu, J., Suen, J. Y., Fraser, J. F., & Wilkinson, D. J. C. (2021). Ethical factors determining ECMO allocation during the COVID‐19 pandemic. *BMC Medical Ethics*, *22*, 70.

14 Wilkinson, D., & Savulescu, J. (2018). Prioritisation and parity: Which disabled infants should be candidates for scarce life‐saving treatment. In D. Wasserman & A. Cureton (Eds.), *Oxford handbook of philosophy and disability* (pp. 669‐692). Oxford University Press.

15 Hordern, op. cit. note 3, p. 19.

16 Nagel, T. (1986). *The view from nowhere*. Oxford University Press.

17 Veatch, R. M. (2012). *Hippocratic, religious and secular medical ethics. The points of conflict.* Georgetown University Press. This is not to deny the point that Hordern makes, that a number of nonreligious values or ethical beliefs are contested and not shared (e.g., endorsement of a particular normative theory), or equally that some so‐called ‘religious’ values can be shared by many who are not explicitly religious (e.g., a belief in human dignity).

18 Gilliat Ray, op. cit. note 1.

19 Hordern, op. cit. note 3, p. 18.

20 Connolly, op. cit. note 10.

